# Androgen Receptor Signaling in Prostate Cancer Genomic Subtypes

**DOI:** 10.3390/cancers15204969

**Published:** 2023-10-13

**Authors:** Mohamed Ali Hussein, Gnanasekar Munirathinam

**Affiliations:** 1 Department of Pharmaceutical Services, Children’s Cancer Hospital Egypt, Cairo 57357, Egypt; mohamedalihussien8554@gmail.com; 2 Department of Biology, School of Sciences and Engineering, American University in Cairo, New Cairo 11835, Egypt; 3 Department of Biomedical Sciences, College of Medicine, University of Illinois, Rockford, IL 61107, USA

## 1. Introduction

Prostate cancer (PCa) constitutes a significant cause of mortality, with over 37,000 new deaths each year. It is also the most commonly diagnosed cancer in older men, with more than 288,000 new cases expected in the United States in 2023 [1]. The androgen receptor (AR) belongs to the nuclear receptor superfamily and acts as a transcription factor that is activated by ligands. In its unbound state, the AR is located in the cytosol and is stabilized by heat-shock proteins. However, when it binds to its ligand, it translocate to the nucleus and binds to a specific DNA sequence known as the androgen response element (ARE) [2]. AR play a crucial role in modulating PCa pathogenesis, such as cell proliferation and disease progression [3]. Resistance to AR-targeted therapy is widespread and multifactorial; in addition, it is the primary driver of the development of a lethal disease status known as castration-resistant prostate cancer (CRPC). Resistance to androgen deprivation therapy (ADT), including surgical castration, radiation therapy, chemotherapy, or anti-androgens such as abiraterone and enzalutamide, usually emerges within 2–3 years after treatment [4]. Several lines of evidence support that PCa disease progression is AR-signaling-dependent, even in the case of resistance to AR-targeted therapy. Various factors have been suggested as potential causes of drug resistance to AR-targeted treatments, including restored AR signals via AR mutations and gene amplification, the increased expression of AR splice variants, an upregulated tumor androgen level, and evaded AR signaling via the activation of glucocorticoid receptor (GR) signaling as a bypass mechanism that may share similarities with AR signaling [2,4,5].

Jillson et al. [6] conducted a thorough review of the existing literature, as well as a meta-analysis, to uncover primary PCa genomic individual variations, as well as co-occurring variations, their interactions with AR signaling, and the effect on responses to AR-targeted therapy. In addition, they identify multiple genomic subtypes of primary PCa, which are essential in categorizing patient responses to AR-targeted therapy. The significance of this review is that it offers a new perspective on how the combined effects of genetic variations that mutually co-occur in early primary PCa could affect the AR signaling axis and, subsequently, the AR gene, which occurs in the later stage during metastatic CRPC, rather than solely focusing on individual variations. In addition, this review provides a newer perspective by discussing how genetic variation could help in stratifying patients and their responses to AR therapy based on several genetic variations, which will decide whether a patient will respond to AR treatment or need other appropriate therapeutic agents; this will pave the way toward a more personalized treatment option according to patients’ genetic makeup variations.

The authors have begun by first investigating the most common individual genetic variations across three cohorts from the TCGA, the MSKCC, and BROAD. After that, they used the available RNA expression data to show the correlation between AR expression levels and individual variations. Those individual variations include ETS transcription factors such as *ERG*, *ETV1*, *ETV4*, and *FLI1* [7], as well as, most importantly, the fusion of *ERG* with the androgen-regulated gene, *TMPRSS2* [8]. It serves by maintaining the de-differentiated status of prostate epithelial cells, which is opposite to AR. However, *ERG* can directly or indirectly regulate AR expression. The meta-analysis highlights the inverse relationship between AR and *ERG*, with the AR-normalized score in the *ERG-TMPRSS2* fusion being near to or below the normal level. The authors also highlight the inconclusive effect of *ERG* fusion-positive PCa in response to AR-targeted therapy, as one study shows that fusion increases survival rates after ADT. In contrast, another study suggests that fusion might elicit a response to high-dose androgen therapy.

In addition, this review delves into discussing *MAP3K7* loss, its role in PCa progression, and how *MAP3K7* loss affects AR activity. This study uncovered that the simultaneous loss of *MAP3K7* and *CHD1* resulted in the upregulation of AR activity. Moreover, *MAP3K7* loss renders PCa cells more susceptible to DNA damage and cell cycle inhibitors by disturbing the homologous recombination process. Another frequent genetic variation is *CHD1* loss, which increases AR activity and the resistance to AR-targeted therapy. This resistance is typically seen in AR antagonists and enzalutamide, but not in the androgen synthesis inhibitor, abiraterone. This suggests that a patient with *CDH1* loss may benefit from androgen withdrawal treatment. These findings align with the results of a meta-analysis that shows that *CHD1* is associated with higher AR scores, but not higher mRNA levels.

The authors further discussed the interplay between AR and mutation in *SPOP*. The authors revealed that a mutation in *SPOP* can lead to an increased expression of its targeted substrate. As AR and their cofactors are among those substrates, a mutation in *SPOP* can result in an increased AR expression level, as well as its cofactors in primary PCa. This positive correlation between mutant *SPOP* and AR suggests that patients with a mutation in *SPOP* might benefit from ADT, as well as having an enhanced sensitivity to HDAC3 inhibitors and BET inhibitors.

Importantly, the authors delve into discussing the inverse relationship between AR and the PI3K pathway and its downstream, such as mutated or amplified *AKT* as well as *PIK3CA* and *PTEN* deletion [9]. *AKT* facilitates phosphorylates and marks AR for degradation, while AR stabilizes the phosphatases that suppress *AKT*, which is consistent with meta-analysis results. Therefore, targeting both AR and PI3K is recommended instead of solely targeting one or the other, which might lead to resistance. Additionally, the authors explored the delicate relationship between AR and *TP53*, emphasizing the pivotal role of *TP53* in ensuring the functioning of AR. Their analysis has confirmed that a lack of *TP53* may result in reduced AR activity, and may potentially impact efficacy in response to chemotherapy.

Moreover, the authors explored the impact of the cell cycle regulators *RB1* and *CDKN1B* on AR activity and expression. Their findings suggest that *RB1* loss results in an increase in AR activity, despite mixed results from meta-analyses that report overall average AR scores. Although *CDKN1B* has not been extensively explored in PCa, the authors uncovered evidence that *CDKN1B* loss is linked to average or above-average AR scores, and observed that *CDKN1B* deletion often occurs alongside other deletions in *MAP3K7* and *CHD1*. In addition, they further discussed the role of *FOXA1* in regulating AR. They demonstrated that *FOXA1* mutation can either increase or decrease AR activity and expression. Nevertheless, the meta-analysis results show a slightly above-average AR activity.

Furthermore, the authors discussed the correlation between AR and DNA repair genes, such as *PARP1*, *ATM*, and *BRCA1/2*. However, increased DNA damage resulted in increased AR expression and activity, and vice versa. *PARP1* and *ATM* deletion are associated with above-average AR activity; in contrast, the association between *BRCA1/2* mutations and AR varies between cohorts. Hence, it is warranted to dual-inhibit AR and the DNA damage pathway to prevent resistance. *MYC* is a well-known oncogene that plays a crucial role in the development of cancer [10]. *MYC* is found to be over-amplified in PCa and can drive the progression of PCa independently, even in the absence of AR. Consistently, the authors revealed the average AR expression and AR activity levels, implying that AR and *MYC* are independent. Consequently, *MYC* amplification can mitigate the effectiveness of AR-targeted therapy.

While individual genetic variation plays a significant role in the progression of PCa, it alone cannot fully explain the complexity of the disease or identify the distinct subtypes that determine a patient’s response to AR-targeted therapy. To address this, the authors have categorized PCa into five different subtypes based on the co-occurrence of at least two individual genetic variations, including *ERG/PTEN/TP53, MAP3K7/CHD1/SPOP, BRCA1/TP53, BRCA2/RB1*, and *ATM/PARP1*, and examined the impact on the AR score and expression level. The first of these subtypes is *MAP3K7/CHD1/SPOP*, in which *MAP3K7* and *CHD1* co-deletion is common in primary PCa and is associated with an increased AR score, enzalutamide resistance, and increased AR splice variants. In addition, patients with lower expression exhibit an aggressive subtype and are associated with relapse after radical prostatectomy. In contrast, the co-occurring *SPOP* mutation with *CHD1* and *MAP3K7* deletion is associated with increased AR activity as well as sensitivity to baritone and DNA-damaging or -repairing agents. Next, the authors discussed the joint co-occurring *ERG* fusion with almost all other mutations, which indicates an early significant event in the development of primary PCa that confers a favorable trait for the progression of the disease. One subtype involves *ERG* fusion with *PTEN* and *TP53*. Although *ERG* and *PTEN* can individually have a negative effect on AR, the co-occurrence of *ERG* fusion and *PTEN* loss restores AR expression and enhances the resistance to the dual inhibition of PI3K and AR. Although *PTEN* loss in combination with a *TP53* mutation is usually linked to promoting resistance to anti-androgen therapy due to the reduced AR expression and lineage plasticity, the co-occurring *ERG* fusion has the opposite effect by restoring AR expression and rendering PCa more sensitive to anti-androgen treatment.

Moreover, the authors have examined the effect of another subtype, including the significant co-loss of *PARP1* and *ATM*. Despite the individual deletion of *PARP1* and *ATM* being associated with increased AR activity and expression, respectively, their co-deletion is associated with both increased AR activity and expression. Moreover, alterations in *BRCA1* and *BRCA2* are mutually exclusive, and commonly occur with various genetic variations, with *BRCA1* tending to occur with *TP53* deletion, resulting in decreased AR activity. In contrast, *BRCA2* occurs with *RB1* loss, resulting in average AR activity and AR expression levels, as well as enhanced resistance to ADT, mediated mainly by the epithelial–mesenchymal transition.

## 2. Conclusions

In this review, the authors investigate the effect of individual genomic variation, the joint effect of common, concomitantly occurring variations on AR activity and expression, and their role in primary PCa progression and responses to various treatment options. They further stratify primary PCa into five distinct subtypes. The authors revealed that individual as well as co-occurring alterations impact AR activity by either increasing activity and promoting survival, decreasing activity and facilitating lineage plasticity, or inducing genomic instability, which can lead to disease progression and affect responses to different treatments. This review presents a new viewpoint regarding the influence of genetic variation on a patient’s response to AR therapy. By pinpointing genetic variations that are significantly linked, it may be feasible to stratify patients and decide whether they will respond to AR treatment or require an alternative therapeutic regimen according to their genetic makeup. This personalized approach has the potential to result in more efficient therapies based on a patient’s genetic makeup.

## References

[1] Siegel R.L., Miller K.D., Wagle N.S., Jemal A. (2023). Cancer statistics, 2023. CA Cancer J. Clin..

[2] Van-Duyne G., Blair I.A., Sprenger C., Moiseenkova-Bell V., Plymate S., Penning T.M. (2023). The androgen receptor. Vitam. Horm..

[3] Messner E.A., Steele T.M., Tsamouri M.M., Hejazi N., Gao A.C., Mudryj M., Ghosh P.M. (2020). The Androgen Receptor in Prostate Cancer: Effect of Structure, Ligands and Spliced Variants on Therapy. Biomedicines.

[4] Shafi A.A., Yen A.E., Weigel N.L. (2013). Androgen receptors in hormone-dependent and castration-resistant prostate cancer. Pharmacol. Ther..

[5] Watson P.A., Arora V.K., Sawyers C.L. (2015). Emerging mechanisms of resistance to androgen receptor inhibitors in prostate cancer. Nat. Rev. Cancer.

[6] Jillson L.K., Yette G.A., Laajala T.D., Tilley W.D., Costello J.C., Cramer S.D. (2021). Androgen Receptor Signaling in Prostate Cancer Genomic Subtypes. Cancers.

[7] Oikawa T., Yamada T. (2003). Molecular biology of the Ets family of transcription factors. Gene.

[8] Wang Z., Wang Y., Zhang J., Hu Q., Zhi F., Zhang S., Mao D., Zhang Y., Liang H. (2017). Significance of the TMPRSS2:ERG gene fusion in prostate cancer. Mol. Med. Rep..

[9] Shorning B.Y., Dass M.S., Smalley M.J., Pearson H.B. (2020). The PI3K-AKT-mTOR Pathway and Prostate Cancer: At the Crossroads of AR, MAPK, and WNT Signaling. Int. J. Mol. Sci..

[10] Stine Z.E., Walton Z.E., Altman B.J., Hsieh A.L., Dang C.V. (2015). MYC, Metabolism, and Cancer. Cancer Discov..

